# Isoflurane titration improves detection of hippocampal lactate by ^1^ H-MRS

**DOI:** 10.1162/imag_a_00305

**Published:** 2024-10-04

**Authors:** Ariel K. Frame, Reza Khazaee, Marc Courchesne, Scott K Wilson, Miranda Bellyou, Alex X. Li, Robert Bartha, Robert C. Cumming

**Affiliations:** Department of Biology, University of Western Ontario, London, ON, Canada; Biotron Integrated Microscopy Facility, University of Western Ontario, London, ON, Canada; Centre for Functional and Metabolic Mapping, Robarts Research Institute, Schulich School of Medicine & Dentistry, University of Western Ontario, London, ON, Canada; Department of Medical Biophysics, Schulich School of Medicine & Dentistry, University of Western Ontario, London, ON, Canada

**Keywords:** lactate, ^1^ H-MRS, isoflurane, hippocampus, lactate dehydrogenase, anesthetic

## Abstract

Lactate has increasingly been recognized as both an important fuel source and a signaling molecule within the brain. Alterations in brain lactate levels are associated with various neurological diseases. Thus, there is great interest in the*in vivo*detection and measurement of cerebral lactate levels in animals used for investigation of normal brain function and models of disease. Proton magnetic resonance spectroscopy (^1^H-MRS) is a non-invasive technique used to measure lactate and other metabolites within the brain. However, lactate can be difficult to detect with conventional^1^H-MRS due to its low abundance and spectral overlap with lipids. In addition, volatile anesthetics used during image acquisition increase lactate production, potentially masking any subtle physiological changes in lactate levels. Here, we made use of a transgenic mouse model in which expression of lactate dehydrogenase A (*Ldha*), the rate-limiting enzyme of lactate production, was induced within cortical and hippocampal neurons. Unexpectedly,^1^H-MRS analysis, under typical isoflurane-induced anesthesia of 4% induction followed by 1.6–2% maintenance, revealed no significant elevation of hippocampal lactate levels in neuronal*Ldha*induction mice compared to control mice. In contrast,^1^H-MRS analysis, using an isoflurane titration protocol in which mice were sequentially exposed to 1.6%, 2%, and then finally 3% isoflurane, revealed significantly higher hippocampal lactate levels in*Ldha*transgenic mice compared to controls. In addition, significantly fewer mice were required to detect differences in lactate levels using the isoflurane titration protocol compared to conventional isoflurane-induced anesthesia. Our findings highlight the importance of controlling for the effects of anesthesia when detecting changes in hippocampal lactate levels*in vivo*and offer a novel protocol for enhanced cerebral lactate detection.

## 1 Introduction

Highly elevated cerebral lactate levels are typically associated with ischemic events (Henriksen et al., 1992;Higuchi et al., 1996;Hyacinthe et al., 2020;Woo et al., 2010). However, numerous studies have demonstrated that under non-ischemic conditions, more subtle changes in lactate play multiple key roles in brain physiology, including synaptic plasticity, long-term potentiation, and memory formation (Bingul et al., 2020;Dembitskaya et al., 2022;Suzuki et al., 2011;Yang et al., 2014). In addition, lactate can function as a signaling molecule within the brain by binding to the G-protein coupled receptor-81 (GPR81), also known as hydroxycarboxylic acid receptor 1 (HCAR1), thereby decreasing neuronal excitability (Ahmed et al., 2010;Barros, 2013;Briquet et al., 2022;Cai et al., 2008;Liu et al., 2009). Altered lactate metabolism has been implicated in various neurological disorders, including Schizophrenia, Attention Deficit Disorder, Alzheimer’s, and Parkinson’s disease (Bonomi et al., 2021;Liguori et al., 2016;Medin et al., 2019;Rowland et al., 2016). Thus, there is great interest in detecting both major alterations in cerebral lactate levels under pathological conditions and subtle alterations in cerebral lactate levels under normal circumstances.

Proton magnetic resonance spectroscopy (^1^H-MRS) is a non-invasive technique for measuring brain metabolites*in vivo*(van der Graaf, 2010). Detection of lactate by^1^H-MRS using a single echo time, which generally appears as a doublet at a frequency of approximately 1.3 ppm, can be difficult due to its low abundance and spectral overlap with lipids, particularly at short echo times. However, increased detection of lactate and suppression of water signal can be achieved using an array of echo times and 2-dimensional J-resolved MRS to detect the J-coupling behavior of lactate (Weaver et al., 2015) or by adjusting the echo time such that the lactate peaks are inverted relative to lipids. MRS studies typically require that animals are anesthetized to prevent any movement during the scanning process. Assessment of lactate by^1^H-MRS can be confounded by elevation of this metabolite following exposure to halogenated volatile anesthetics such as isoflurane and sevoflurane (Boretius et al., 2013;Horn & Klein, 2010;Makaryus et al., 2011;L. Zhang et al., 2022). Moreover,^1^H-MRS measurement revealed that lactate levels in the brain are specifically raised while other macromolecules remain relatively stable when isoflurane dose was increased from 1% to 2%, despite alterations to diffusion of intracellular metabolites (Valette et al., 2007). Several lines of evidence suggest that isoflurane interferes with mitochondrial function (Fedorov et al., 2023;Kishikawa et al., 2018;Y. Zhang et al., 2012), possibly by inhibiting complex I (Zimin et al., 2018), thereby promoting NADH accumulation which, in turn, can lead to increased lactate production to maintain ATP levels. Therefore, it is critical to carefully control for the concentration and duration of exposure of isoflurane when assessing cerebral lactate levels by^1^H-MRS. Moreover, this is especially important when attempting to measure non-pathological changes in cerebral lactate which may be of a small magnitude.

The astrocyte-to-neuron lactate shuttle hypothesis posits that astrocytes produce and release lactate, which is then transported and oxidized by adjacent neurons to fuel synaptic transmission, a highly energy demanding process (Barros & Deitmer, 2010;Pellerin & Magistretti, 1994). Many studies corroborate the seminal 2011 study (Suzuki et al., 2011) showing the astrocyte-neuron lactate shuttle fuels cognition. However, elevated local lactate associated with neuronal activity (Hu & Wilson, 1997;Prichard et al., 1991;Sappey-Marinier et al., 1992) may also be explained by neuronal glycolysis and lactate release (Díaz-García et al., 2017;Ivanov et al., 2014). To assess the role of neuronal-produced lactate on hippocampal-dependent cognitive function, we generated a transgenic mouse with inducible expression of lactate dehydrogenase A (*Ldha*) within cortical and hippocampal neurons. Because LDHA is predominantly biased towards the conversion of pyruvate to lactate (Kaplan et al., 1968;Vesell, 1965), we anticipated that neuronal*Ldha*induction mice would promote an increase in hippocampal lactate levels. Surprisingly, hippocampal lactate levels did not differ in neuronal*Ldha*induction mice compared with control mice when measured using^1^H-MRS analysis under standard isoflurane anesthetic conditions. Because standard anesthetic conditions employ high level isoflurane exposure, which can artificially elevate cerebral lactate levels (Boretius et al., 2013;Horn & Klein, 2010;Makaryus et al., 2011;L. Zhang et al., 2022), we hypothesized that progressively increasing isoflurane concentrations over a prolonged period of time would allow the brain to adapt and lessen the artificial elevation of lactate levels typically caused by high isoflurane levels initially used for induction of anesthesia. Using a modified anesthetic protocol in which mice were progressively exposed to increasing concentrations of isoflurane over a longer period of time revealed a significant increase in hippocampal lactate levels in neuronal*Ldha*induction compared to control mice. Our findings indicate that conventional^1^H-MRS protocols using isoflurane at concentrations that are not systematically controlled may be inadequate for detection of cerebral lactate under non-pathological conditions. Use of a protocol whereby isoflurane concentration and duration of exposure is precisely monitored, such as the isoflurane titration method utilized in this study, is recommended to improve detection of subtle increases of hippocampal lactate by^1^H-MRS.

## 2 Materials and Methods

### 2.1 Mice

**Fig. 1.**
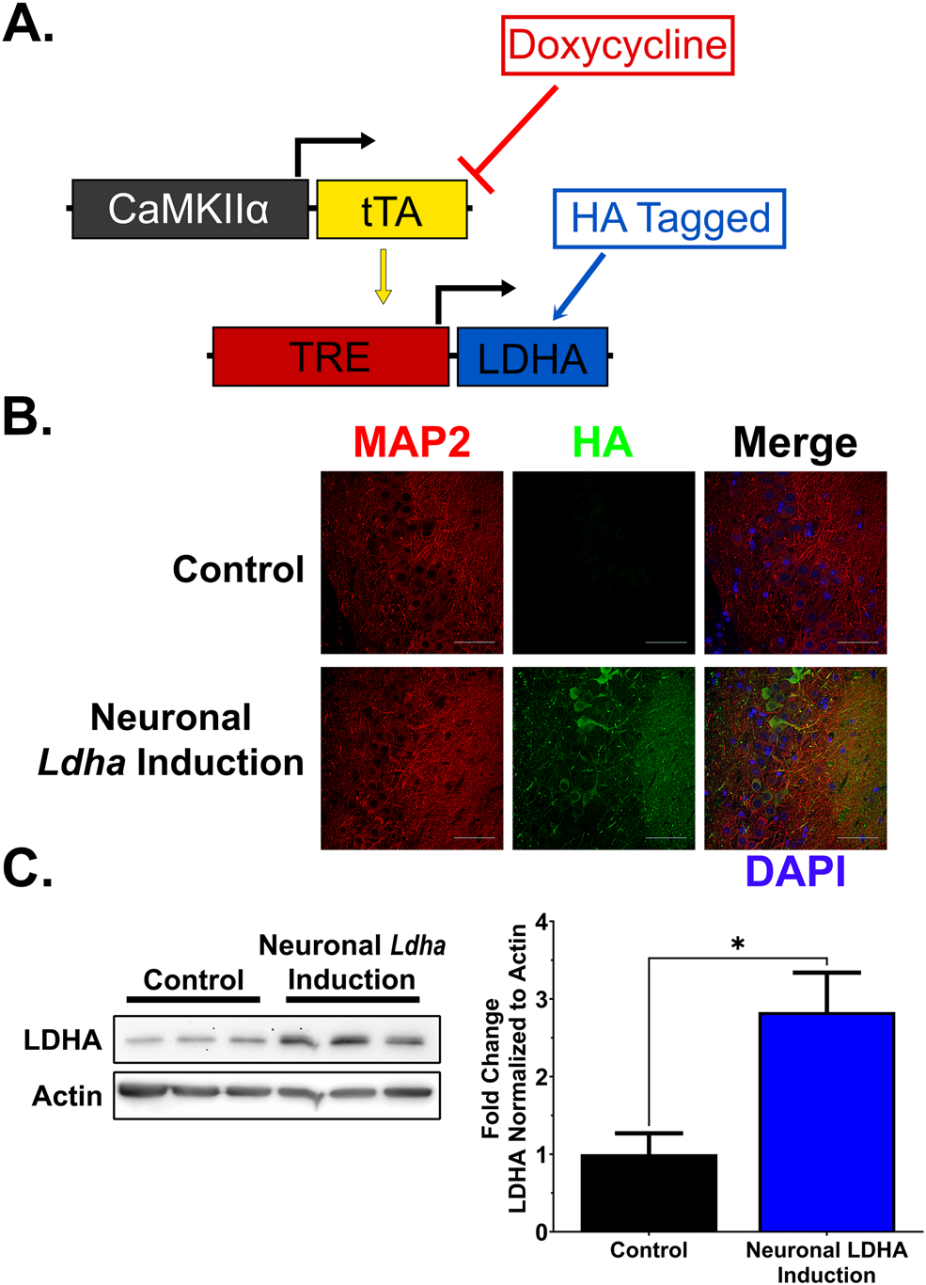
Generation of an inducible neuronal*Ldha*induction mouse model. (A) Schematic outlining the two genetic constructs combined for neuronal*Ldha*induction. Control mice lack one or both constructs. Induction was initiated in adulthood by withdrawal of dietary doxycycline. (B) Immunofluorescence images of hippocampal CA2 region verifying production of HA-tagged LDHA in neuronal*Ldha*induction mice. (C) Western blot analysis of hippocampal protein extracts showing increased LDHA in neuronal*Ldha*induction mice compared with control (*t*(4) = 3.183, * =*p*<0.05). n = 3. Comparison made using unpaired*t*-test.

All animal procedures were conducted in accordance with Canadian Council of Animal Care guidelines and protocols (2011-079 and 2020-112) approved by the animal care committee (ACC) of Western University. Male mice were housed in shoebox cages under standard conditions, including 22–25°C temperature control, 12 h light-dark cycle, and ad libitum access to chow diet (Teklad, 2018, Envigo) and water. Expression of hemagglutanin (HA) tagged*Ldha*in neurons was achieved by crossing neuronal tetracycline transactivator driver mice (calcium-calmodulin-dependent kinase II (CaMKIIα) promoter; CaMKII-tTA; 007004) (Mayford et al., 1996) obtained from The Jackson Laboratory and mice containing a transgene with a tetracycline response element (TRE) promoter driving HA-tagged*Ldha*expression (TRE-LDHA) produced in house (RRID:MGI:7645158), as previously described (Frame et al., 2024). The TetOff system (Gossen & Bujard, 1992;Schönig & Bujard, 2013) utilized in these transgenic mice was restricted to adulthood by withdrawal of doxycycline (S3888, Bio-Serv) from the diet during the post-weaning period (Fig. 1A). Male mice were scanned using^1^H-MRS at 7–9 months of age and euthanized at 9.5 months of age with carbon dioxide for brains to be harvested. Prior to brains being removed, mice were cardiac perfused with phosphate buffered saline (PBS) containing phenylmethylsulfonyl fluoride (PMSF) protease inhibitor (P7626, Millipore Sigma) and sodium orthovanadate phosphatase inhibitor (S6508, Millipore Sigma).

### 2.2 Proton magnetic resonance spectroscopy

**Fig. 2.**
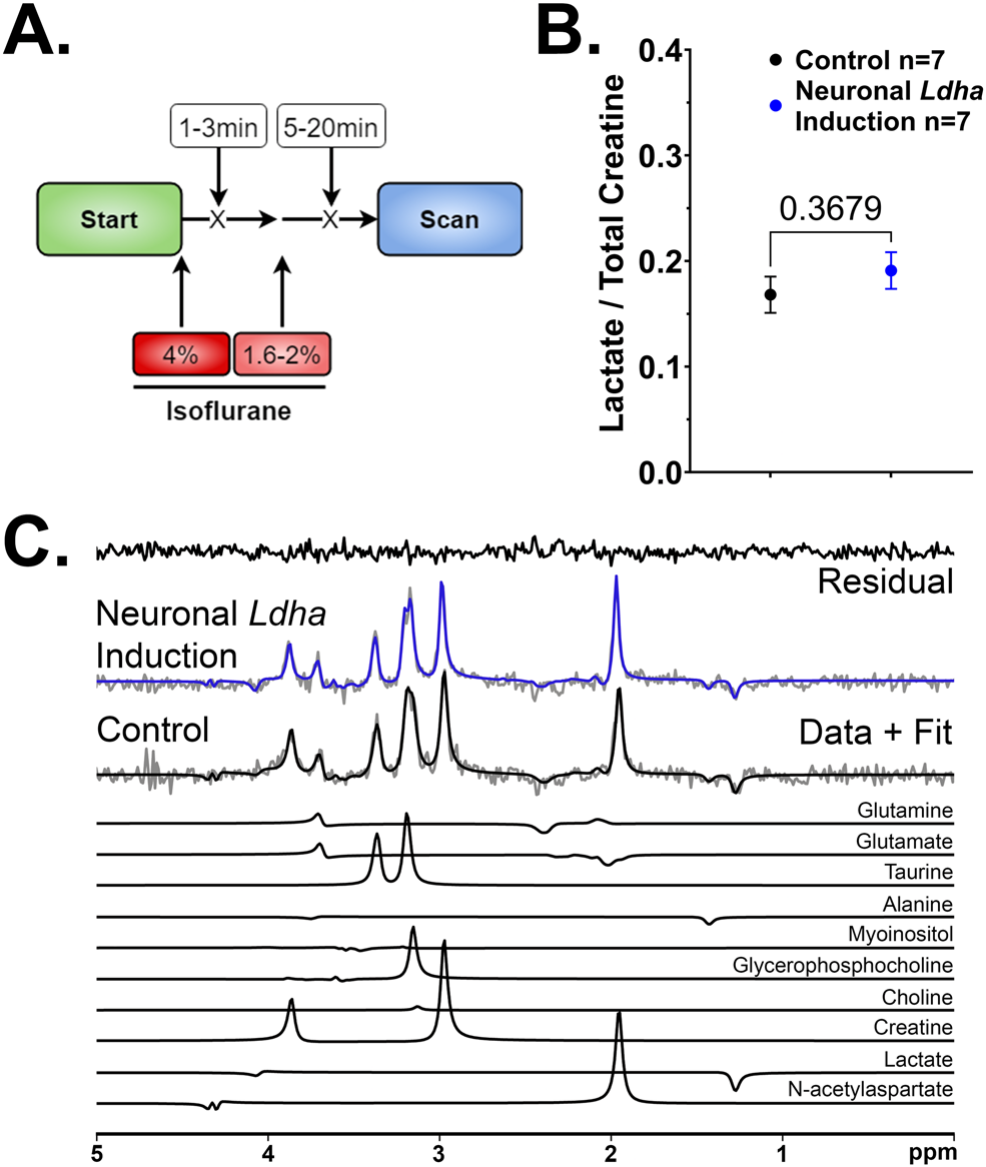
Hippocampal lactate levels do not differ between neuronal Ldha induction and control mice using a standard isoflurane protocol. (A) Schematic outlining the standard isoflurane protocol used for^1^H-MRS. (B) Quantification of hippocampal lactate/creatine levels using a standard isoflurane protocol for^1^H-MRS revealed no change between neuronal*Ldha*induction and control mice (*t*(12) = 0.9357,*p*= 0.3679). Comparison made using unpaired*t*-test. (C) Representative MRS spectra acquired*in-vivo*in each group along with the model fit and residual. Individual major metabolite components, including lactate which is a distinctly inverted peak at 1.3 ppm when using an echo time of 136 ms.

**Fig. 3.**
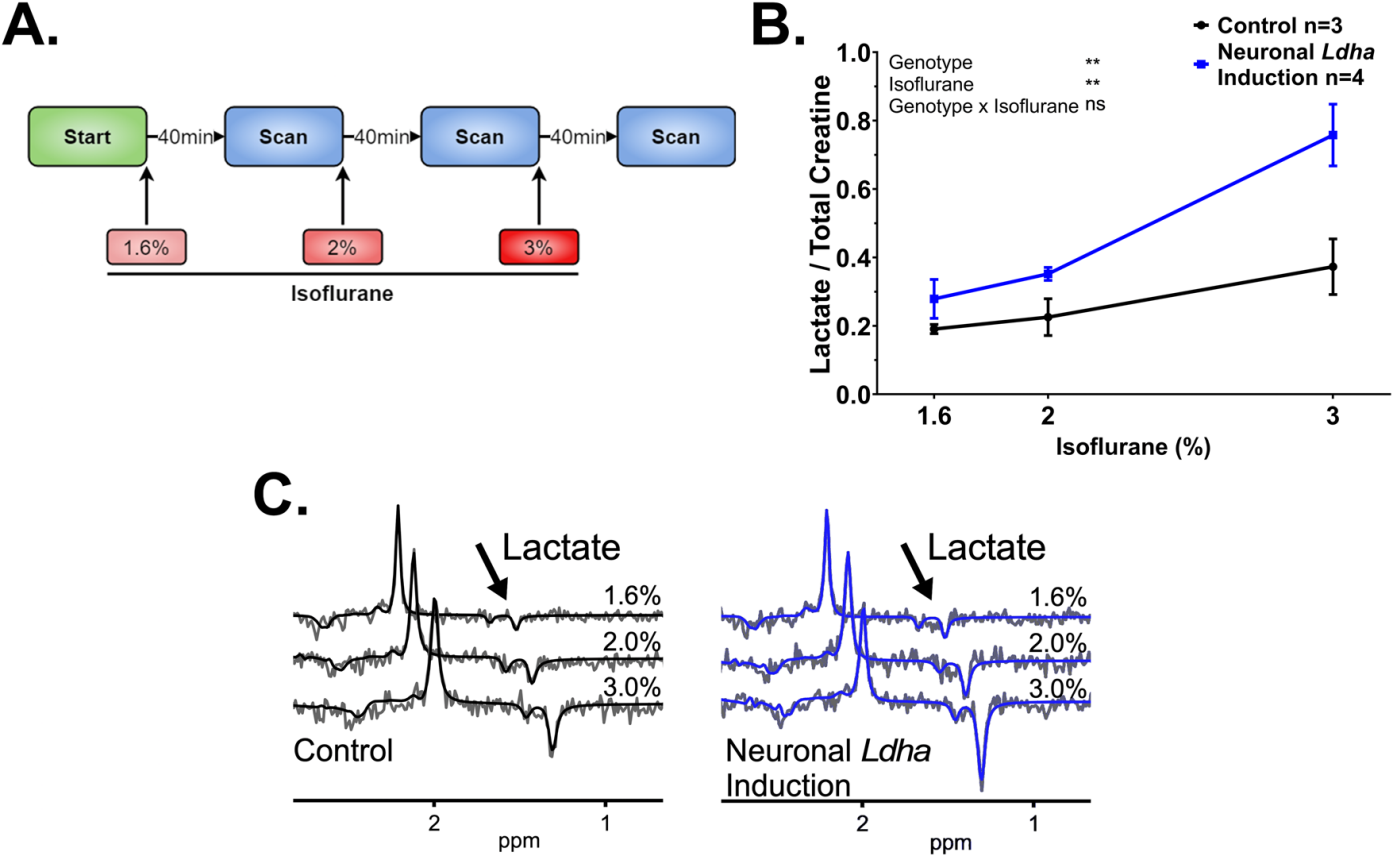
Titration of isoflurane concentration reveals an increase in hippocampal lactate levels in neuronal*Ldha*induction mice. (A) Schematic outlining the isoflurane titration protocol used for^1^H-MRS. (B) Quantification of hippocampal lactate/creatine using an isoflurane titration protocol for^1^H-MRS shows an increase in neuronal*Ldha*induction compared to control mice (genotype effect:*F*(1,15) = 15.47, ** =*p*<0.01). Comparison made using a mixed-effects model with Geisser-Greenhouse correction, fixed effects presented in each graph, and Šídák’s multiple comparisons test. (C) Changes in lactate levels observed in the magnetic resonance spectrum in a control and neuronal*Ldha*induction mouse as a function of isoflurane level (1.6%, 2%, and 3%).

During scans using a standard isoflurane protocol, isoflurane concentration was started at 4% and then maintained between 1.5% and 2.5% (Fig. 2A) with an oxygen flow rate of 1−1.5 L/min through a custom-built nose cone. For scans where the isoflurane was titrated, isoflurane concentration was maintained for 40 min prior to each scan and changed sequentially from 1.6% to 2% to 3% (Fig. 3A). Animal temperature was monitored with a rectal temperature probe, and respiration was monitored with a pneumatic pillow connected to a pressure transducer that was placed on the thoracic region. Body temperature was maintained at 36.9−37.1°C throughout imaging by blowing warm air over the animal using a model 1,025 small-animal monitoring and gating system (SA Instruments Inc., Stony Brook, NY, USA). Magnetic resonance imaging (MRI) experiments were performed on a 9.4-T/31-cm small animal MRI scanner (Agilent, Palo Alto, CA, USA) interfaced to a Bruker Avance III HD console (Bruker BioSpin Corp, Billerica, MA) and equipped with a 6-cm gradient coil of 1,000 mT/m strength, running Paravision-6 software at the Centre for Functional and Metabolic Mapping located within the Robarts Research Institute at the University of Western Ontario. A Varian 30-mm millipede volume radiofrequency coil was used for data collection. At the beginning of each scan, coronal T2-weighted anatomical images were acquired using a TurboRARE2D pulse sequence (16 averages, 31 slices with slice thickness of 0.5 mm, FOV = 19.2 × 19.2 mm^2^, matrix size = 128 × 128, in-plane resolution = 0.15 × 0.15 mm^2^, TE = 40 ms, TR = 5.0 s, echo spacing = 10 ms, and rare factor = 8). For planning the MRS voxel, a 2 × 6 × 3 mm^3^voxel was positioned over both hippocampi (Supplementary Fig. S1) for MRS data acquisition using the semi-LASER (Semi-Localization by Adiabatic Selective Refocusing) sequence that provides localized spectra from rectangular voxels selected with a sequence of a 90° excitation pulse and two pairs of 180° adiabatic refocusing pulses (TR/TE = 5,000/136 ms) (Garwood & DelaBarre, 2001). A water spectrum was acquired first (8 acquisitions), followed by a metabolite spectrum with water suppression (128 acquisitions). Metabolite spectra (including N-acetylaspartate, lactate, alanine, glutamate, glutamine, creatine (total creatine, including creatine and phosphocreatine), taurine, choline, glycerophosphocholine, and myoinositol) were post-processed using eddy current correction (ECC) and fitted using the fitMAN software (Bartha et al., 1999) incorporated into a graphical user interface written in the IDL (Interactive Data Language) programming language (Kassem & Bartha, 2003) to determine the amplitude of lactate and creatine resonances. The lactate over creatine ratio was calculated by dividing the sum of each amplitude resonance after correction for the T2 relaxation time-constant of lactate (161) and creatine (104) using the formula: as previously described (Wong et al., 2018).

### 2.3 Immunofluorescence microscopy

The left hemisphere of each brain was post-fixed in 4% paraformaldehyde (15713, Electron Microscopy Sciences) in phosphate buffer for 3 days, stored in ethanol, embedded in paraffin wax, and sectioned with a rotary microtome (RM2055, Leica Biosystems) at a 5 µm thickness sagittally until the hippocampal dentate gyrus was visible. Sections were baked onto glass slides (12-550-15, ThermoFisher Scientific), deparaffinized, and subjected to heat-induced epitope retrieval with 10 mM sodium citrate buffer pH 6.0 (C7129, Millipore Sigma). Background autofluorescence was quenched with UV exposure, 1 mg/ml sodium borohydride, and TrueBlack (23007, Biotium). Sections were blocked with goat anti-mouse affinity-purified fab fragment antibodies (115-007-003, Jackson ImmunoResearch Inc.; 1:40) and Background Sniper (BS966, Biocare Medical). Primary antibodies were applied at 4°C overnight, including mouse anti-HA.11 epitope tag (901513, BioLegend; 1:500), rabbit anti-MAP2 (ab32454, Abcam; 1:1,000), and secondary antibodies were applied at room temperature for 40 min, including goat anti-mouse Alexa Fluor 568 (A11031, Thermofisher Scientific; 1:500) and goat anti-rabbit Alexa Fluor 647 (A21244, ThermoFisher Scientific; 1:500). 4’,6-diamidino-2-phenylindole (DAPI; D1306, ThermoFisher Scientific; 1:300) was used to counterstain for nuclei. Fluorescence microscopy images were taken of each section using a Nikon ECLIPSE Ti2-E microscope system with plan apochromatic lambda dry 20 x objective (MRD00205, Nikon Instruments) and a monochrome backside illuminated scientific complementary metal–oxide–semiconductor image sensor (pco.edge 4.2 bi, Excelitas Technologies). The excitation and emission wavelengths (nm) used for detection of fluorescence from Alexa Fluor 568 were 554 and 595, and from Alexa Fluor 647 were 635 and 681. NIS-Elements AR (RRID:SCR_014329, Nikon Instruments) with the 2D deconvolution module was used to automatically remove out-of-focus light.

### 2.4 Western blot analysis

Protein was extracted from the right hemisphere of each brain using a lysis buffer containing 250 mM sucrose (S0389, Millipore Sigma), 50 mM tris (BP152, ThermoFisher Scientific), 25 mM potassium chloride (P4504, Millipore Sigma), 1% triton X (T9284, Millipore Sigma), 0.5 mM PMSF (P7626, Millipore Sigma), 1X Halt™ protease inhibitor cocktail (87786, ThermoFisher Scientific), and 0.1 mM sodium orthovanadate (S6508, Millipore Sigma) and quantified using a detergent compatible assay (5000111, Bio-Rad). Protein extracts, alongside a protein standard (1610373, Bio-Rad), were resolved by sodium dodecyl sulfate–polyacrylamide gel electrophoresis (SDS-PAGE) and transferred to a polyvinylidene fluoride (PVDF) membrane blot. Blots were blocked with 1% w/v bovine serum albumin (BSA) and 3% w/v milk, probed with primary antibodies at 4°C overnight, including rabbit anti-LDHA (2012, Cell Signaling; 1:1,000) and mouse anti-actin (sc-47778, Santa Cruz Biotechnology; 1:2,000), and secondary antibodies at room temperature for 1 h, including goat anti-mouse (AP130P, Millipore Sigma; 1:10,000) and goat anti-rabbit (AP132P, Millipore Sigma; 1:10,000) conjugated with horse radish peroxidase (HRP). Chemiluminescent signal was detected using Forte Western HRP substrate (WBLUF, Millipore Sigma), imaged using a ChemiDoc XRS imaging system (170-8070, Bio-Rad), and quantified using Image Lab software (RRID:SCR_014210, Bio-Rad). Western blot HA band intensity was standardized to actin band intensity for quantification.

### 2.5 Statistical analysis

Data were analyzed statistically and visualized using GraphPad Prism version 10.0.0 (153) (RRID:SCR_002798). Data were presented as mean ± SEM with statistical comparisons described in each figure legend. Power analysis was conducted using the pwr package (Champely, 2018) in R version 4.3.1 (2023-06-16 ucrt, RRID:SCR_001905) using RStudio (RRID:SCR_000432) (R Core Team, 2015;RStudio Team, 2020).

## 3 Results

### 3.1 Generation of an inducible neuronal *Ldha* induction mouse model

To assess the effects of altered lactate levels in the brain by^1^H-MRS, we generated a transgenic mouse line with a hemagglutinin (HA)-tagged murine*Ldha*cDNA under the control of the tetracycline response element promoter (TRE-LDHA). This line was subsequently crossed to transgenic mice expressing the tetracycline transactivator (tTA) under regulatory control of the neuron-specific calcium/calmodulin-dependent kinase IIα promoter (CaMKIIα-tTA), a driver highly expressed in the cortex and hippocampus (Fig. 1A). In dual transgenic mice, induced expression of*Ldha*in CNS neurons was achieved following the removal of doxycycline, a tetracycline analogue, from the diet of weaned mice. Induction of LDHA was confirmed by both immunofluorescence microscopy (Fig. 1B) and western blotting (Fig. 1C).

### 3.2 Hippocampal lactate levels do not differ between neuronal *Ldha* induction and control mice using a standard isoflurane protocol

To determine if neuronal induction of*Ldha*expression promoted increased lactate production in the hippocampus, a key brain structure required for learning and memory, we performed^1^H-MRS analysis under standard anesthesia conditions using isoflurane at a concentration of 4% for induction followed by 1.6–2% for maintenance (Fig. 2A). Surprisingly, no significant elevation of hippocampal lactate/creatine levels was detected in neuronal induction*Ldha*mice compared to control mice (Fig. 2B). Typically, the time between isoflurane induction followed by a maintenance level of isoflurane is generally not systematically controlled in most studies. Therefore, the absence of altered hippocampal lactate levels in*Ldha*induction mice relative to controls may have potentially been attributed to confounding effects related to the concentration and duration of isoflurane exposure used for induction and maintenance of anesthesia.

### 3.3 Sequential increase in isoflurane level reveals changes in hippocampal lactate concentration

A previous study byHorn and Klein (2010)demonstrated that continuous isoflurane administration promotes a progressive increase in cerebral lactate levels that eventually reaches a plateau. In addition, lactate levels increase in a manner proportional to isoflurane concentration (Boretius et al., 2013;Horn & Klein, 2010). Mice exposed to 1.75% isoflurane followed by a period of no anesthetic show a pronounced drop in cerebral lactate levels (Boretius et al., 2013). Interestingly, following re-exposure to isoflurane, lactate levels once again increased but not to the same level as during the initial exposure (Boretius et al., 2013). In addition, numerous studies have shown that isoflurane preconditioning leads to adaptive responses in the brain (McMurtrey & Zuo, 2010). Thus, we hypothesized that progressively increasing isoflurane concentrations over a prolonged period of time would allow the brain to adapt and lessen the artificial elevation of lactate levels typically caused by high isoflurane levels initially used for induction of anesthesia.

To test this hypothesis, we measured hippocampal lactate/creatine in neuronal*Ldha*induction mice with an altered^1^H-MRS protocol by systematically titrating the isoflurane concentration from 1.6%, 2%, and 3% (Fig. 3A). This protocol specifies that scanning occurs 40 min after each isoflurane concentration increase to allow for lactate levels to stabilize (Fig. 3A). As expected, we found that increasing the isoflurane concentration resulted in an increase in hippocampal lactate/creatine levels in all mice (Fig. 3B). However, by employing a sequential elevation in the concentration of isoflurane used for anesthesia revealed a significant increase in hippocampal lactate/creatine in neuronal*Ldha*induction mice compared with control mice (Fig. 3B). Importantly, the absence of an isoflurane by genotype effect indicates that these two genotypes do not differ in their susceptibility to isoflurane induction of lactate (Fig. 3B).

Normalization of lactate to creatine eliminates two problems associated with measurement of absolute values, including variance introduced by measurement of tissues within the voxel that are required to distinguish cerebrospinal fluid and tissue fractions, and error introduced by scaling to water in the voxel. Nonetheless, normalizing to creatine could potentially be problematic if this metabolite is also affected by isoflurane. Thus, we quantified absolute levels of both lactate and creatine under standard and isoflurane titration concentrations. Similar to lactate/creatine measurements, standard isoflurane conditions revealed no differences in absolute lactate levels between control and*Ldha*induction mice (Supplementary Fig. S2A). As observed in lactate/creatine measurements, the progressive increase in isoflurane concentration resulted in elevated absolute levels of lactate in all mice (Supplementary Fig. S2B). However,*Ldha*induction mice showed a trend of increased absolute lactate levels similar to the lactate/creatine measurements (Supplementary Fig. S2B). In contrast, absolute levels of creatine did not change under standard isoflurane conditions (Supplementary Fig. S2C) or with the isoflurane titration protocol (Supplementary Fig. S2D) for either control or*Ldha*induction mice. Thus, the genotype specific elevation of lactate/creatine levels in*Ldha*induction mice is attributed to changes in lactate but not creatine.

These data provide strong evidence that isoflurane titration is a valuable method for measuring increases in hippocampal lactate by^1^H-MRS in genetically manipulated mice.

## 4 Discussion

In the present study, we demonstrate the utility of systematically controlling isoflurane concentration used for anesthesia during measurement of^1^H-MRS-based hippocampal lactate levels in mice. We show here that mice genetically manipulated to promote increased capacity for lactate production within CNS neurons exhibit increased hippocampal lactate/creatine levels detectable by^1^H-MRS but only when isoflurane concentration is titrated. The utility of this method may be expanded to studies seeking to detect cerebral lactate under circumstances where small changes are expected. For example, animal models of neurodegenerative disease which may have progressive accumulation of cerebral lactate (Hagihara et al., 2024) may employ isoflurane titration during^1^H-MRS to detect small increases in lactate that develop earlier. Furthermore, the increased power afforded by our isoflurane titration method also reduced the number of animals required to detect changes in hippocampal lactate. If we intended on designing a study using standard anesthetic conditions to achieve an 80% chance of detecting an increase in hippocampal lactate in our neuronal*Ldha*induction mice with the effect size we observed in this study (Fig. 2B; Cohen’s d = 0.63), then a power analysis (α = 0.05, β = 0.2) indicates that we would have required over 63 animals per condition. In contrast, we were able to detect an increase in hippocampal lactate in our neuronal*Ldha*induction mice applying our isoflurane titration method using less than 5 animals per condition. Therefore, isoflurane titration during cerebral lactate measurement with^1^H-MRS may facilitate more humane studies, according to the reduction principle of human experimental technique (Russell & Burch, 1959) while increasing the probability of detecting changes that would have otherwise been undiscovered under standard anesthesia conditions.

In this study, lactate levels were standardized to a reference peak representing creatine and phosphocreatine combined as a measure of total creatine. In the past, studies have shown that total creatine in the brain is largely unaffected by isoflurane level used for anesthesia (Boretius et al., 2013;Söbbeler et al., 2018). A relatively low concentration of isoflurane (1.75%) compared to no isoflurane caused lactate/creatine levels to increase greatly (533%) (Boretius et al., 2013). Here, we showed that absolute levels of creatine do not change with increasing concentrations of isoflurane. This suggests that elevated lactate/creatine levels with increasing isoflurane exposure are primarily due to changes in lactate and not creatine.

One could seek alternative methods of assessing lactate in the brain*in vivo*while avoiding potential confounds introduced by anesthesia. For example, a neuromuscular blocking agent could be administered to mice without anesthesia such that MRS can be performed while mice are incapable of movement. However, the use of a neuromuscular blocking agent could also cause confounding effects. For example, does the chosen neuromuscular blocking agent impact CNS lactate? Does a lack of anesthesia allow the animal to perceive the MRS procedure as stressful and leave the animal susceptible to stress-induced CNS lactate alterations? Moreover, local animal care committees, following institutional and governmental guidelines that differ around the world, may not consider it ethical to administer a potentially stressful procedure without anesthetic. For example, the Canadian Council on Animal Care (CCAC) suggests that anesthesia should be used during potentially stressful procedures (CCAC, 2019). Therefore, while alternatives to anesthesia may avoid confounding anesthesia related effects or can provide a non-anesthetized baseline*in vivo*measurement, ethical concerns should be also considered. In light of the ubiquity in which volatile anesthetics are used during MRS procedures, ideally customizing a protocol that retains anesthesia while permitting the ability to discriminate between the effects of anesthesia versus physiological or pathological changes would provide the most utility.

In recent years, there have been newly developed adaptations to MRS methods for detection of lactate, such as deuterium metabolic imaging (DMI) (De Feyter et al., 2018;Flatt et al., 2021), quantitative exchanged-label turn-over MRS (qMRS) (Rich et al., 2020), diffusion-weighted magnetic resonance spectroscopy (DW-MRS) (Ligneul et al., 2019), chemical shift imaging (CSI) (Brender et al., 2019), double selective multiple quantum filter technique (SelMQC) (Pickup et al., 2008), and lactate chemical exchange saturation transfer LATEST (DeBrosse et al., 2016). To our knowledge, systematic titration of anesthetic concentration has not been adopted as a method for lactate detection using any of the aforementioned newer methods. Moreover, these studies are similarly susceptible to isoflurane-induced changes in cerebral lactate. Therefore, adding isoflurane titration to any MRS protocol offers a safe and effective strategy for reducing isoflurane-induced lactate changes regardless of the detection method. Overall, future use of the simple modification to MRS protocols we describe here has the potential to greatly increase the power and detection capabilities when measuring cerebral lactate levels in animal models.

## 5 Limitations

This study provides a relatively simple protocol that can enhance the use of isoflurane anesthesia during MRS assessment of brain lactate levels in mice. However, alternative methods of conducting MRS without the use of anesthesia could eliminate or reduce artificial anesthesia-induced effects on brain metabolites, particularly lactate (Ferris, 2022;Madularu et al., 2017). MRS without anesthesia is limited by potential confounding effects that come with increased stress and required acclimation. Thus, until MRS analysis of awake mice has been perfected, and as long as isoflurane remains the most common method of anesthesia for MRS, the protocol described here should still retain utility.

## Supplementary Material

Supplementary Material

## Data Availability

All raw data and statistical analyses are available on the Borealis data repository (https://doi.org/10.5683/SP3/ROJAYY).

## References

[b1] Ahmed , K. , Tunaru , S. , Tang , C. , Müller , M. , Gille , A. , Sassmann , A. , Hanson , J. , & Offermanns , S. ( 2010 ). An autocrine lactate loop mediates insulin-dependent inhibition of lipolysis through GPR81 . Cell Metabolism , 11 ( 4 ), 311 – 319 . 10.1016/j.cmet.2010.02.012 20374963

[b2] Barros , L. F. ( 2013 ). Metabolic signaling by lactate in the brain . Trends in Neurosciences , 36 ( 7 ), 396 – 404 . 10.1016/j.tins.2013.04.002 23639382

[b3] Barros , L. F. , & Deitmer , J. W. ( 2010 ). Glucose and lactate supply to the synapse . Brain Research Reviews , 63 ( 1–2 ), 149 – 159 . 10.1016/j.brainresrev.2009.10.002 19879896

[b4] Bartha , R. , Drost , D. J. , & Williamson , P. C. ( 1999 ). Factors affecting the quantification of short echoin-vivo1H MR spectra: Prior knowledge, peak elimination, and filtering . NMR in Biomedicine , 12 ( 4 ), 205 – 216 . 10.1002/(SICI)1099-1492(199906)12:4<205::AID-NBM558>3.0.CO;2-1 10421912

[b5] Bingul , D. , Kalra , K. , Murata , E. M. , Belser , A. , & Dash , M. B. ( 2020 ). Persistent changes in extracellular lactate dynamics following synaptic potentiation . Neurobiology of Learning and Memory , 175 , 107314 . 10.1016/j.nlm.2020.107314 32961277 PMC7655607

[b6] Bonomi , C. G. , De Lucia , V. , Mascolo , A. P. , Assogna , M. , Motta , C. , Scaricamazza , E. , Sallustio , F. , Mercuri , N. B. , Koch , G. , & Martorana , A. ( 2021 ). Brain energy metabolism and neurodegeneration: Hints from CSF lactate levels in dementias . Neurobiology of Aging , 105 , 333 – 339 . 10.1016/j.neurobiolaging.2021.05.011 34171631

[b7] Boretius , S. , Tammer , R. , Michaelis , T. , Brockmöller , J. , & Frahm , J. ( 2013 ). Halogenated volatile anesthetics alter brain metabolism as revealed by proton magnetic resonance spectroscopy of mice in vivo . NeuroImage , 69 , 244 – 255 . 10.1016/j.neuroimage.2012.12.020 23266699

[b8] Brender , J. R. , Kishimoto , S. , Merkle , H. , Reed , G. , Hurd , R. E. , Chen , A. P. , Ardenkjaer-Larsen , J. H. , Munasinghe , J. , Saito , K. , Seki , T. , Oshima , N. , Yamamoto , K. , Choyke , P. L. , Mitchell , J. , & Krishna , M. C. ( 2019 ). Dynamic imaging of glucose and lactate metabolism by 13C-MRS without hyperpolarization . Scientific Reports , 9 ( 1 ), 3410 . 10.1038/s41598-019-38981-1 30833588 PMC6399318

[b9] Briquet , M. , Rocher , A.-B. , Alessandri , M. , Rosenberg , N. , de Castro Abrantes , H. , Wellbourne-Wood , J. , Schmuziger , C. , Ginet , V. , Puyal , J. , Pralong , E. , Daniel , R. T. , Offermanns , S. , & Chatton , J.-Y. ( 2022 ). Activation of lactate receptor HCAR1 down-modulates neuronal activity in rodent and human brain tissue . Journal of Cerebral Blood Flow & Metabolism , 42 ( 9 ), 1650 – 1665 . 10.1177/0271678X221080324 35240875 PMC9441721

[b10] Cai , T.-Q. , Ren , N. , Jin , L. , Cheng , K. , Kash , S. , Chen , R. , Wright , S. D. , Taggart , A. K. P. , & Waters , M. G. ( 2008 ). Role of GPR81 in lactate-mediated reduction of adipose lipolysis . Biochemical and Biophysical Research Communications , 377 ( 3 ), 987 – 991 . 10.1016/j.bbrc.2008.10.088 18952058

[b11] CCAC . ( 2019 ). CCAC Guidelines: Mice . Canadian Council on Animal Welfare . https://ccac.ca/

[b12] Champely , S. ( 2018 ). pwr: Basic Functions for Power Analysis (R package version 1.2-2). http://cran.r-project.org/package=pwr

[b13] DeBrosse , C. , Nanga , R. P. R. , Bagga , P. , Nath , K. , Haris , M. , Marincola , F. , Schnall , M. D. , Hariharan , H. , & Reddy , R. ( 2016 ). Lactate chemical exchange saturation transfer (LATEST) imaging in vivo a biomarker for LDH activity . Scientific Reports , 6 , 19517 . 10.1038/srep19517 26794265 PMC4726389

[b14] De Feyter , H. M. , Behar , K. L. , Corbin , Z. A. , Fulbright , R. K. , Brown , P. B. , McIntyre , S. , Nixon , T. W. , Rothman , D. L. , & de Graaf , R. A. ( 2018 ). Deuterium metabolic imaging (DMI) for MRI-based 3D mapping of metabolism in vivo . Science Advances , 4 ( 8 ), eaat7314 . 10.1126/sciadv.aat7314 30140744 PMC6105304

[b15] Dembitskaya , Y. , Piette , C. , Perez , S. , Berry , H. , Magistretti , P. J. , & Venance , L. ( 2022 ). Lactate supply overtakes glucose when neural computational and cognitive loads scale up . Proceedings of the National Academy of Sciences of the United States of America , 119 ( 47 ), e2212004119 . 10.1073/pnas.2212004119 36375086 PMC9704697

[b16] Díaz-García , C. M. , Mongeon , R. , Lahmann , C. , Koveal , D. , Zucker , H. , & Yellen , G. ( 2017 ). Neuronal stimulation triggers neuronal glycolysis and not lactate uptake . Cell Metabolism , 26 ( 2 ), 361.e4 – 374.e4 . 10.1016/j.cmet.2017.06.021 28768175 PMC5559896

[b17] Fedorov , A. , Lehto , A. , & Klein , J. ( 2023 ). Inhibition of mitochondrial respiration by general anesthetic drugs . Naunyn-Schmiedeberg’s Archives of Pharmacology , 396 ( 2 ), 375 – 381 . 10.1007/s00210-022-02338-9 36385685 PMC9832080

[b18] Ferris , C. F. ( 2022 ). Applications in awake animal magnetic resonance imaging . Frontiers in Neuroscience , 16 , 1 – 17 . 10.3389/fnins.2022.854377 PMC9017993 35450017

[b19] Flatt , E. , Lanz , B. , Pilloud , Y. , Capozzi , A. , Lerche , M. H. , Gruetter , R. , & Mishkovsky , M. ( 2021 ). Measuring glycolytic activity with hyperpolarized [2H7, U-13C6] D-glucose in the naive mouse brain under different anesthetic conditions . Metabolites , 11 ( 7 ), 413 . 10.3390/metabo11070413 34201777 PMC8303162

[b20] Frame , A. K. , Sinka , J. L. , Courchesne , M. , Muhammad , R. A. , Grahovac-Nemeth , S. , Bernards , M. A. , Bartha , R. , & Cumming , R. C. ( 2024 ). Altered neuronal lactate dehydrogenase A expression affects cognition in a sex- and age-dependent manner . IScience , 27 ( 7 ), 110342 . 10.1016/j.isci.2024.110342 . 39055955 PMC11269950

[b21] Garwood , M. , & DelaBarre , L. ( 2001 ). The return of the frequency sweep: Designing adiabatic pulses for contemporary NMR . Journal of Magnetic Resonance , 153 ( 2 ), 155 – 177 . 10.1006/jmre.2001.2340 11740891

[b22] Gossen , M. , & Bujard , H. ( 1992 ). Tight control of gene expression in mammalian cells by tetracycline-responsive promoters . Proceedings of the National Academy of Sciences of the United States of America , 89 ( 12 ), 5547 – 5551 . 10.1073/pnas.89.12.5547 1319065 PMC49329

[b23] Hagihara , H. , Shoji , H. , Hattori , S. , Sala , G. , Takamiya , Y. , Tanaka , M. , Ihara , M. , Shibutani , M. , Hatada , I. , Hori , K. , Hoshino , M. , Nakao , A. , Mori , Y. , Okabe , S. , Matsushita , M. , Urbach , A. , Katayama , Y. , Matsumoto , A. , Nakayama , K. I. , … Miyakawa , T. ( 2024 ). Large-scale animal model study uncovers altered brain pH and lactate levels as a transdiagnostic endophenotype of neuropsychiatric disorders involving cognitive impairment . ELife , 12 , 1 – 27 . 10.7554/eLife.89376.3 PMC10965225 38529532

[b24] Henriksen , O. , Gideon , P. , Sperling , B. , Olsen , T. S. , Jørgensen , H. S. , & Arlien-Søborg , P. ( 1992 ). Cerebral lactate production and blood flow in acute stroke . Journal of Magnetic Resonance Imaging , 2 ( 5 ), 511 – 517 . 10.1002/jmri.1880020508 1392243

[b25] Higuchi , T. , Fernandez , E. J. , Maudsley , A. A. , Shimizu , H. , Weiner , M. W. , & Weinstein , P. R. ( 1996 ). Mapping of lactate and N-acetyl-L-aspartate predicts infarction during acute focal ischemia: In vivo 1H magnetic resonance spectroscopy in rats . Neurosurgery , 38 ( 1 ), 121 – 130 . 10.1097/00006123-199601000-00030 8747960

[b26] Horn , T. , & Klein , J. ( 2010 ). Lactate levels in the brain are elevated upon exposure to volatile anesthetics: A microdialysis study . Neurochemistry International , 57 ( 8 ), 940 – 947 . 10.1016/j.neuint.2010.09.014 20933036

[b27] Hu , Y. , & Wilson , G. S. ( 1997 ). A temporary local energy pool coupled to neuronal activity: Fluctuations of extracellular lactate levels in rat brain monitored with rapid-response enzyme-based sensor . Journal of Neurochemistry , 69 ( 4 ), 1484 – 1490 . 10.1046/j.1471-4159.1997.69041484.x 9326277

[b28] Hyacinthe , J. N. , Buscemi , L. , Lê , T. P. , Lepore , M. , Hirt , L. , & Mishkovsky , M. ( 2020 ). Evaluating the potential of hyperpolarised [1-13C] L-lactate as a neuroprotectant metabolic biosensor for stroke . Scientific Reports , 10 , 5507 . 10.1038/s41598-020-62319-x 32218474 PMC7099080

[b29] Ivanov , A. , Malkov , A. E. , Waseem , T. , Mukhtarov , M. , Buldakova , S. , Gubkina , O. , Zilberter , M. , & Zilberter , Y. ( 2014 ). Glycolysis and oxidative phosphorylation in neurons and astrocytes during network activity in hippocampal slices . Journal of Cerebral Blood Flow & Metabolism , 34 ( 3 ), 397 – 407 . 10.1038/jcbfm.2013.222 24326389 PMC3948126

[b30] Kaplan , N. O. , Everse , J. , & Admiraal , J. ( 1968 ). Significance of substrate inhibition of dehydrogenases . Annals of the New York Academy of Sciences , 151 ( 1 ), 400 – 412 . 10.1111/j.1749-6632.1968.tb11903.x 4304804

[b31] Kassem , M. N. E. , & Bartha , R. ( 2003 ). Quantitative proton short-echo-time LASER spectroscopy of normal human white matter and hippocampus at 4 Tesla incorporating macromolecule subtraction . Magnetic Resonance in Medicine , 49 ( 5 ), 918 – 927 . 10.1002/mrm.10443 12704775

[b32] Kishikawa , J.-I , Inoue , Y. , Fujikawa , M. , Nishimura , K. , Nakanishi , A. , Tanabe , T. , Imamura , H. , & Yokoyama , K. ( 2018 ). General anesthetics cause mitochondrial dysfunction and reduction of intracellular ATP levels . PLoS One , 13 ( 1 ), e0190213 . 10.1371/journal.pone.0190213 29298324 PMC5752027

[b33] Ligneul , C. , Palombo , M. , Hernández-Garzón , E. , Carrillo-de Sauvage , M. A. , Flament , J. , Hantraye , P. , Brouillet , E. , Bonvento , G. , Escartin , C. , & Valette , J. ( 2019 ). Diffusion-weighted magnetic resonance spectroscopy enables cell-specific monitoring of astrocyte reactivity in vivo . NeuroImage , 191 , 457 – 469 . 10.1016/j.neuroimage.2019.02.046 30818026

[b34] Liguori , C. , Chiaravalloti , A. , Sancesario , G. M. G. , Stefani , A. , Sancesario , G. M. G. , Mercuri , N. B. , Schillaci , O. , & Pierantozzi , M. ( 2016 ). Cerebrospinal fluid lactate levels and brain [18F]FDG PET hypometabolism within the default mode network in Alzheimer’s disease . European Journal of Nuclear Medicine and Molecular Imaging , 43 ( 11 ), 2040 – 2049 . 10.1007/s00259-016-3417-2 27221635

[b35] Liu , C. , Wu , J. , Zhu , J. , Kuei , C. , Yu , J. , Shelton , J. , Sutton , S. W. , Li , X. , Yun , S. J. , Mirzadegan , T. , Mazur , C. , Kamme , F. , & Lovenberg , T. W. ( 2009 ). Lactate inhibits lipolysis in fat cells through activation of an orphan G-protein-coupled receptor, GPR81 . Journal of Biological Chemistry , 284 ( 5 ), 2811 – 2822 . 10.1074/jbc.M806409200 19047060

[b36] Madularu , D. , Mathieu , A. P. , Kumaragamage , C. , Reynolds , L. M. , Near , J. , Flores , C. , & Rajah , M. N. ( 2017 ). A non-invasive restraining system for awake mouse imaging . Journal of Neuroscience Methods , 287 , 53 – 57 . 10.1016/j.jneumeth.2017.06.008 28634149 PMC5790991

[b37] Makaryus , R. , Lee , H. , Yu , M. , Zhang , S. , Smith , S. D. , Rebecchi , M. , Glass , P. S. , & Benveniste , H. ( 2011 ). The metabolomic profile during isoflurane anesthesia differs from propofol anesthesia in the live rodent brain . Journal of Cerebral Blood Flow & Metabolism , 31 ( 6 ), 1432 – 1442 . 10.1038/jcbfm.2011.1 21266982 PMC3130322

[b38] Mayford , M. , Bach , M. E. , Huang , Y.-Y. , Wang , L. , Hawkins , R. D. , & Kandel , E. R. ( 1996 ). Control of memory formation through regulated expression of a CaMKII transgene . Science , 274 ( 5293 ), 1678 – 1683 . 10.1126/science.274.5293.1678 8939850

[b39] McMurtrey , R. J. , & Zuo , Z. ( 2010 ). Isoflurane preconditioning and postconditioning in rat hippocampal neurons . Brain Research , 1358 , 184 – 190 . 10.1016/j.brainres.2010.08.015 20709037 PMC2949531

[b40] Medin , T. , Medin , H. , Hefte , M. B. , Storm-Mathisen , J. , & Bergersen , L. H. ( 2019 ). Upregulation of the lactate transporter monocarboxylate transporter 1 at the blood-brain barrier in a rat model of attention-deficit/hyperactivity disorder suggests hyperactivity could be a form of self-treatment . Behavioural Brain Research , 360 , 279 – 285 . 10.1016/j.bbr.2018.12.023 30550949

[b41] Pellerin , L. , & Magistretti , P. J. ( 1994 ). Glutamate uptake into astrocytes stimulates aerobic glycolysis: A mechanism coupling neuronal activity to glucose utilization . Proceedings of the National Academy of Sciences of the United States of America , 91 ( 22 ), 10625 – 10629 . http://www.ncbi.nlm.nih.gov/pubmed/7938003 7938003 10.1073/pnas.91.22.10625 PMC45074

[b42] Pickup , S. , Lee , S. , Mancuso , A. , & Glickson , J. D. ( 2008 ). Lactate imaging with Hadamard‐encoded slice‐selective multiple quantum coherence chemical‐shift imaging . Magnetic Resonance in Medicine , 60 ( 2 ), 299 – 305 . 10.1002/mrm.21659 18666110 PMC2701382

[b43] Prichard , J. , Rothman , D. , Novotny , E. , Petroff , O. , Kuwabara , T. , Avison , M. , Howseman , A. , Hanstock , C. , & Shulman , R. ( 1991 ). Lactate rise detected by 1H NMR in human visual cortex during physiologic stimulation . Proceedings of the National Academy of Sciences of the United States of America , 88 ( 13 ), 5829 – 5831 . 10.1073/pnas.88.13.5829 2062861 PMC51971

[b44] R Core Team . ( 2015 ). R: A language and environment for statistical computing (4.3.1 (2023-06-16 ucrt)). R Foundation for Statistical Computing . http://www.r-project.org/

[b45] Rich , L. J. , Bagga , P. , Wilson , N. E. , Schnall , M. D. , Detre , J. A. , Haris , M. , & Reddy , R. ( 2020 ). 1H magnetic resonance spectroscopy of 2H-to-1H exchange quantifies the dynamics of cellular metabolism in vivo . Nature Biomedical Engineering , 4 ( 3 ), 335 – 342 . 10.1038/s41551-019-0499-8 PMC7071956 31988460

[b46] Rowland , L. M. , Pradhan , S. , Korenic , S. , Wijtenburg , S. A. , Hong , L. E. , Edden , R. A. , & Barker , P. B. ( 2016 ). Elevated brain lactate in schizophrenia: A 7 T magnetic resonance spectroscopy study . Translational Psychiatry , 6 ( 11 ), e967 – e967 . 10.1038/tp.2016.239 27898072 PMC5290358

[b47] RStudio Team . ( 2020 ). RStudio: Integrated Development for R (2023.06.2 Build 561). RStudio, PBC . http://www.rstudio.com/

[b48] Russell , W. M. S. , & Burch , R. L. B. ( 1959 ). The Principles of Humane Experimentation Technique . Methuen & Co Ltd . https://search.worldcat.org/title/1486173

[b49] Sappey-Marinier , D. , Calabrese , G. , Fein , G. , Hugg , J. W. , Biggins , C. , & Weiner , M. W. ( 1992 ). Effect of photic stimulation on human visual cortex lactate and phosphates using 1H and 31 P magnetic resonance spectroscopy . Journal of Cerebral Blood Flow & Metabolism , 12 ( 4 ), 584 – 592 . 10.1038/jcbfm.1992.82 1618937

[b50] Schönig , K. , & Bujard , H. ( 2013 ). Generating conditional mouse mutants via tetracycline-controlled gene expression . In M. H. Hofker & J. van Deursen (Eds.), Transgenic mouse methods and protocols (pp. 69 – 104 ). Humana Press . 10.1385/1-59259-340-2:69 12357963

[b51] Söbbeler , F. J. , Carrera , I. , Pasloske , K. , Ranasinghe , M. G. , Kircher , P. , & Kästner , S. B. R. ( 2018 ). Effects of isoflurane, sevoflurane, propofol and alfaxalone on brain metabolism in dogs assessed by proton magnetic resonance spectroscopy (1H MRS) . BMC Veterinary Research , 14 ( 1 ), 69 . 10.1186/s12917-018-1396-1 29506576 PMC5839062

[b52] Suzuki , A. , Stern , S. A. , Bozdagi , O. , Huntley , G. W. , Walker , R. H. , Magistretti , P. J. , & Alberini , C. M. ( 2011 ). Astrocyte-neuron lactate transport is required for long-term memory formation . Cell , 144 ( 5 ), 810 – 823 . 10.1016/j.cell.2011.02.018 21376239 PMC3073831

[b53] Valette , J. , Guillermier , M. , Besret , L. , Hantraye , P. , Bloch , G. , & Lebon , V. ( 2007 ). Isoflurane strongly affects the diffusion of intracellular metabolites, as shown by 1H nuclear magnetic resonance spectroscopy of the monkey brain . Journal of Cerebral Blood Flow & Metabolism , 27 ( 3 ), 588 – 596 . 10.1038/sj.jcbfm.9600353 16788716

[b54] van der Graaf , M. ( 2010 ). In vivo magnetic resonance spectroscopy: Basic methodology and clinical applications . European Biophysics Journal , 39 ( 4 ), 527 – 540 . 10.1007/s00249-009-0517-y 19680645 PMC2841275

[b55] Vesell , E. S. ( 1965 ). Lactate dehydrogenase isozymes: Substrate inhibition in various human tissues . Science , 150 ( 3703 ), 1590 – 1593 . 10.1126/science.150.3703.1590 5866654

[b56] Weaver , K. E. , Richards , T. L. , Logsdon , R. G. , McGough , E. L. , Minoshima , S. , Aylward , E. H. , Kleinhans , N. M. , Grabowski , T. J. , McCurry , S. M. , & Teri , L. ( 2015 ). Posterior cingulate lactate as a metabolic biomarker in amnestic mild cognitive impairment . BioMed Research International , 2015 , 1 – 13 . 10.1155/2015/610605 PMC4568343 26417597

[b57] Wong , D. , Schranz , A. L. , & Bartha , R. ( 2018 ). Optimized in vivo brain glutamate measurement using long-echo-time semi-LASER at 7 T . NMR in Biomedicine , 31 ( 11 ), e4002 . 10.1002/nbm.4002 30144183

[b58] Woo , C.-W. , Lee , B. S. , Kim , S. T. , & Kim , K.-S. ( 2010 ). Correlation between lactate and neuronal cell damage in the rat brain after focal ischemia: An in vivo 1H magnetic resonance spectroscopic (1H-MRS) study . Acta Radiologica , 51 ( 3 ), 344 – 350 . 10.3109/02841850903515395 20144147

[b59] Yang , J. , Ruchti , E. , Petit , J.-M. , Jourdain , P. , Grenningloh , G. , Allaman , I. , & Magistretti , P. J. ( 2014 ). Lactate promotes plasticity gene expression by potentiating NMDA signaling in neurons . Proceedings of the National Academy of Sciences of the United States of America , 111 ( 33 ), 12228 – 12233 . 10.1073/pnas.1322912111 25071212 PMC4143009

[b60] Zhang , L. , Mao , H. , Yan , J. , Cheng , Y. , Xue , Z. , Qiu , Z. , & Jiang , H. ( 2022 ). Sevoflurane enhances brain glycolysis and lactate production in aged marmosets . British Journal of Anaesthesia , 129 ( 3 ), e63 – e66 . 10.1016/j.bja.2022.05.035 35787801

[b61] Zhang , Y. , Xu , Z. , Wang , H. , Dong , Y. , Shi , H. N. , Culley , D. J. , Crosby , G. , Marcantonio , E. R. , Tanzi , R. E. , & Xie , Z. ( 2012 ). Anesthetics isoflurane and desflurane differently affect mitochondrial function, learning, and memory . Annals of Neurology , 71 ( 5 ), 687 – 698 . 10.1002/ana.23536 22368036 PMC3942786

[b62] Zimin , P. I. , Woods , C. B. , Kayser , E. B. , Ramirez , J. M. , Morgan , P. G. , & Sedensky , M. M. ( 2018 ). Isoflurane disrupts excitatory neurotransmitter dynamics via inhibition of mitochondrial complex I . British Journal of Anaesthesia , 120 ( 5 ), 1019 – 1032 . 10.1016/j.bja.2018.01.036 29661379 PMC6200108

