# Supplementary material for: Isoflurane titration improves detection of hippocampal lactate by 1 H-MRS
Source: Imaging Neurosci (Camb). 2024 Oct 4;2:imag-2-00305. doi: 10.1162/imag_a_00305 (PMC12290798; doi:10.1162/imag_a_00305)
Supplement: Supplementary Material [file imag_a_00305-supp.pdf]

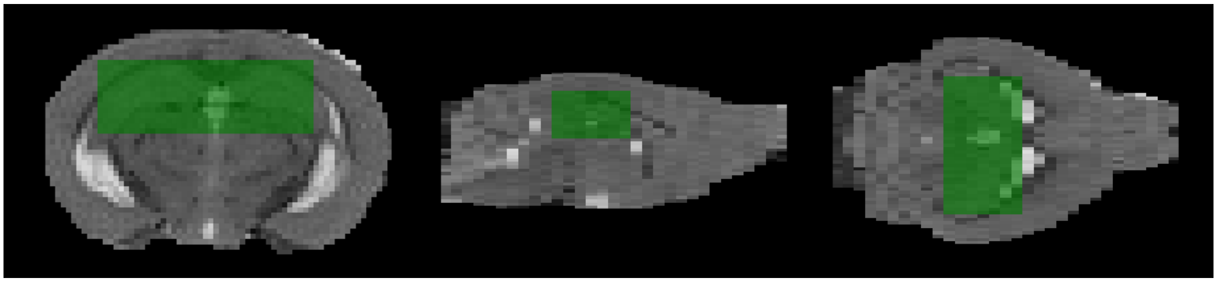

Figure S1. Hippocampi voxel overlay of mouse brain extracted T2 weighted anatomical image.
This image shows where the 2 x 6 x 3 mm^3 voxel was positioned such that it covers both hippocampi for MRS data acquisition.

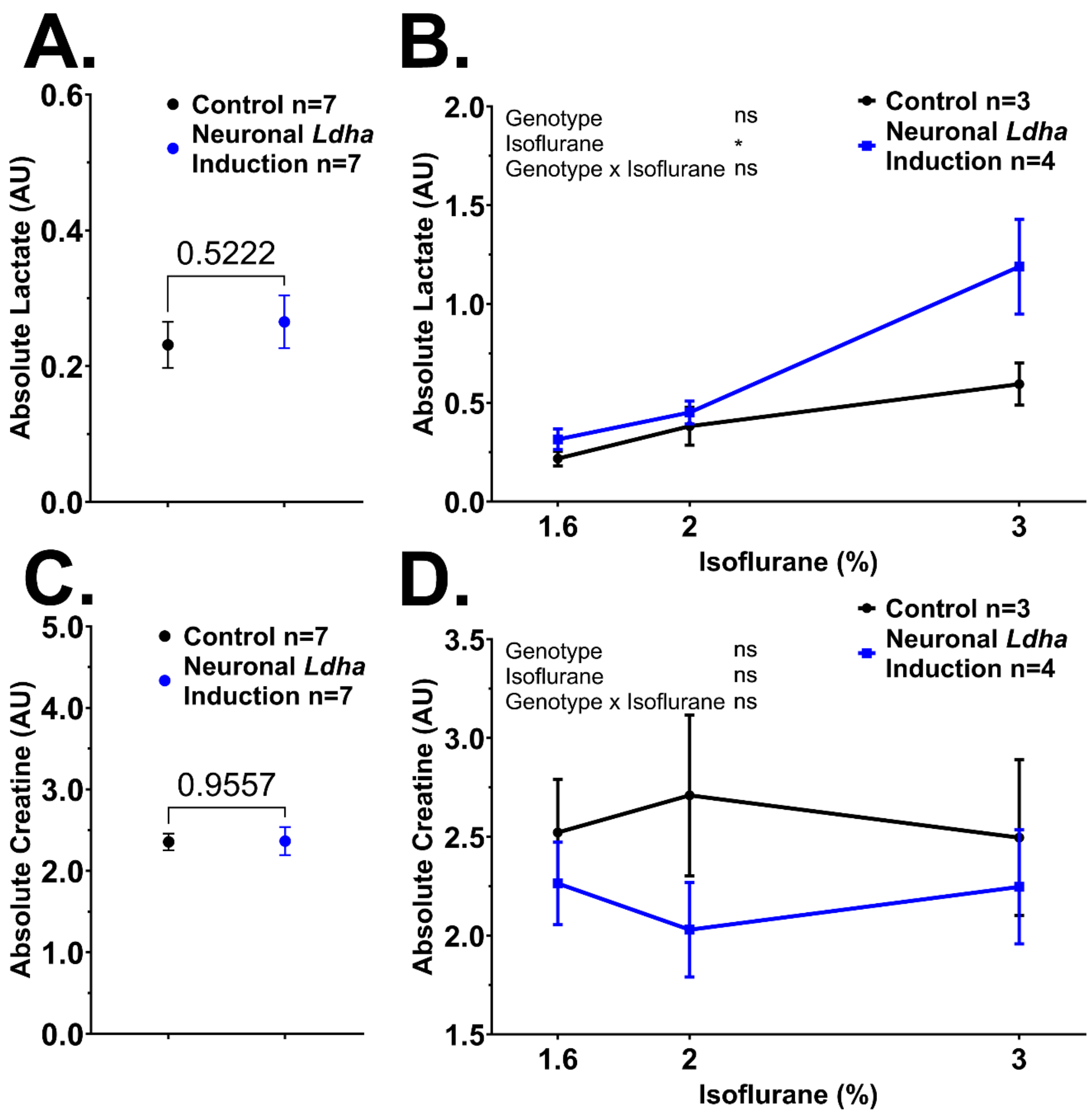

Figure S2. Quantification of absolute levels of lactate and creatine within the hippocampus under standard isoflurane anesthesia or with progressive increases in isoflurane concentration.

A. Quantification of absolute lactate levels in the hippocampus using a standard isoflurane protocol for ^{1}H-MRS revealed no change between neuronal *Ldha* induction and control mice ($t(12)=0.6592$, $p=0.5222$). **B.** Quantification of absolute lactate levels in the hippocampus using an isoflurane titration protocol for ^{1}H-MRS shows a similar trend to quantification using lactate/creatine in Figure 3B (genotype effect: $F(1,5)=4.616$, $p=0.0844$) with an increase in lactate as a function of isoflurane level (1.6%, 2%, and 3%) evident (isoflurane effect: $F(1.109, 5.545)=13.66$, $p=0.0109$). **C.** Quantification of absolute hippocampal creatine levels using a standard isoflurane protocol for ^{1}H-MRS revealed no change between neuronal *Ldha* induction and control mice ($t(12)=0.05671$, $p=0.9557$). **D.** Quantification of absolute hippocampal creatine levels using an isoflurane titration protocol for ^{1}H-MRS revealed no differences between genotypes or as a function of isoflurane level (1.6%, 2%, and 3%). Comparisons for A. and C. made using unpaired t-tests. Comparisons for B. and C. made using a mixed-effects model with Geisser-Greenhouse correction, fixed effects presented in each graph, and Šídák's multiple comparisons test.
